# State Board of Health

**Published:** 1875-12

**Authors:** J. P. Logan, H. H. Carlton, G. F. Cooper

**Affiliations:** Committee; Committee; Committee


ATLANTA

MEDICAL AND SURGICAL JOURNAL.

VOL. XIII.] DECEMBER—1875. [No. 9.

### Original Communications.

### STATE BOARD OF HEALTH.

### Report of Special Committee Upon the Most Effectual Means of Preventing Small-Pox in Georgia.

*To the State Board of Health:*

Though we have been disappointed in obtaining such general statistics belonging to our own State as would exhibit beyond question, in tabular form, the pressing necessity for more stringent laws, and the exertion of much greater effort, for the suppression of small-pox, there is no lack of evidence even in the meagre reports we have been able to gather of the recent ravages of this disease in Georgia; and especially is there no difficulty in drawing material from the reports of investigations in other countries and States to which we are no exception, except in a greater disregard of sanitary regulations in this, as well as many other particulars.

We shall not attempt to present any new contribution to the science of this subject, but simply to offer, in a brief way, such facts as are supposed to be pertinent to the objects of this board, and which we can claim as *positive* and *reliable*, and with which we can go before our people and legislative bodies *demanding*, in the name of humanity, the interposition of a strong hand to arrest the wholesale destruction attendant upon the march of this fell destroyer.

There are two propositions which may be confidently stated in regard to the subject intrusted to our consideration, of such a character as to excite amazement when viewed in connection with the continued existence of and fatality from small-pox: First, variola, or small-pox, is one of the most loathsome, highly contagious, and fatal diseases known to ancient or modern times. Secondly, there is no disease in regard to which the means of prevention are so certainly established.

To these might probably be added another statement, applicable to many localities, viz: that its ravages exhibited a greater aggregate mortality than from any other disease; but, as we have no access at present to the vital statistics bearing upon this subject, and as we are proposing to deal with no doubtful propositions, it is sufficient to say that we are constantly subjected to a recurrence of the disease, and to a *proportionate* mortality probably larger than from any other, when occurring in unprotected persons, and especially when, as frequently occurs, we have, in addition to its ordinary highly contagious character, an epidemic influence prevailing, favorable to its rapid and general extension.

The offensive character of the disease during its active stages, and especially the disfiguring and destructive results in cases of recovery, would alone make it to be greatly dreaded; but when we consider, in addition, its highly contagious character, (but few persons escaping its influence when exposed,) and especially the excessive mortality attending it, remedies for its prevention cannot be too highly estimated.

And yet, with the loathsome and horrible features of the disease, its highly contagious character rendering it almost absolutely certain of propagation among exposed and unprotected persons, and the invariable fearful mortality attending its progress, and the equally certain fact that it can be totally prevented by proper precautions, no single year passes without a reign of terror in one or more communities of our State consequent upon the announcement of small-pox, with its necessarily attendant apprehension of risk in exposure, of contracting the disease, and the strong liability in each case of a fatal result: sundering, in many instances to the disgrace of humanity, the strongest and most intimate ties, not only of friendship, but those still more sacred —connected with blood and kindred. And all this, simply, from the most extraordinary negligence of, and indifference to, the use of means within the reach of every individual, which are *absolute* in the power to control and destroy the contagion, upon which the disease depends for its propagation.

We do not hesitate to assert that, in all the range of sanitary science, there is nothing so certain as the protective power of *thorough vaccination* in preventing the extension of small-pox. Strange, indeed, to the most familiar ears, is such an announcement in connection with the daily reports of the desolation of civilized and even intelligent communities from this disease. Only so late as within the last year we have the following reports from intelligent and reliable physicians of the counties of Coweta and Oglethorpe, in this State, detailing the facts connected with the visitation of small-pox in each of those counties, affording not only an illustration of what is likely to occur in any other section at any moment, but also an *example* of what has occurred almost annually in some one or more sections of our State, the particulars of which we have been unable to obtain. Dr. J. G. Earnest, of the city of Newnan, Coweta county, Georgia, kindly writes:

NEWNAN, GA., August 30, 1875.

*J. P. Logan, M.D., Chairman of Special Committee of State Board of Health:*

The following statement will cover most of the facts in reference to the epidemic of small-pox that occurred here last winter. I am sorry that it has to be made without definite notes, and may be defective in reference to one or two of the dates.

Early in October a gentleman returned from a trip to the Eastern cities, and in a few days was taken with varioloid. The attack was very mild, and he would not believe it to be a dangerous malady. He was on the streets, and saw quite a number of people daily. Soon after his recovery, his son, a boy of twelve, who was supposed to have had varioloid several years previous, was attacked with small-pox that proved confluent, and ended fatally on the eleventh or twelfth day. The next case was a man æt. about forty-five, who was attacked December 2, with fever, cephalalgia and violent pain in the back. After a few days he had an eruption, said to have closely resembled roseola, purple spots appearing afterward *under* the skin. About this time he began to have hemorrhages from the mucous membranes, and died exhausted on the seventh day of the attack. Subsequent events I think justify the belief that this was one of those cases designated by Sydenham as "hemorrhagic small-pox." The facts in the foregoing cases have been gathered from the statements of others. Ten days (December 18) after the death of the last mentioned case, I was called to see his child, an infant at the breast, who had suffered thirty-six hours with a burning fever. On the following morning a papular eruption appeared on the lips, spreading gradually over the face and body, until it covered the entire form. As the eruption came out the fever abated. The eruption was developed very regularly until the child was encased in a perfect shield from head to foot, and died on the ninth day.

As soon as the eruption made its appearance on the child, the other members of the family were vaccinated. The family was constituted then of the mother, æt. about thirty, a maiden aunt, æt. forty-five, a boy four years old and a servant girl, (mulatto), æt. twelve years. They were vaccinated thoroughly. On the following day after the vaccination, however, the mother, the maiden aunt and servant girl were attacked with fever, headache and backache. On the third day of the fever the eruption made its appearance on the mother, coming out copiously on the head and face, but very sparsely on the limbs and trunk. There was some trouble with the throat when the secondary fever came on, but not sufficient to prevent the swallowing of gruel, etc. On the ninth day of the eruption, the kidneys ceased to act, and although remedies were freely administered, their function was not resumed. The bladder was repeatedly examined and found empty. The patient died the eleventh day after the eruption made its appearance, with symptoms of uræmic poisoning. (The nurse states that a small quantity of urine was passed just before she expired.) A feature that, so far as I know was unique, was that the eruption continued coming out until the day of her death, each day bringing out a new instalment of papulæ.

The case of the maiden aunt need not be sketched. We only notice the same trouble with the throat and kidneys during the course of the disease, which terminated fatally on the thirteenth day. It should also be stated that on the day before her death she passed some blood from the bowels.

The case of the colored girl was one of fearful interest. The third day after her fever set in she had a few papulæ on the lips and cheek, mingled with about an equal number of petechiæ. She was delirious after the first twelve hours, and did not entirely regain her consciousness when the eruption made its appearance, nor did the fever disappear, but only slightly abated. The petechiæ enlarged, and formed purple splotches beneath the skin that disappeared on pressure. The papulæ passed into the characteristic vesicles, although there were not exceeding twenty or thirty perhaps on her. On the third day of the eruption she commenced to bleed at the nose. This was followed by vomiting blood, soon after she passed "quite a quantity of blood" (nurse's statement) from the bladder, and over a quart from the bowels, dying comatose that night.

On January 6, 1875, an epileptic girl, æt. seventeen, was attacked, and died of confluent small-pox January 14.

The little four year old boy, belonging to the first mentioned family was promptly affected by vaccination, and escaped the disease.

Out of the eight cases but one had ever been vaccinated, and he alone survived.

Pardon me for saying, in conclusion, that I believe vaccination to be a *never failing and perfect protection from variola, where it has been practiced to complete saturation*—that is, until it will not "take" again. I do not believe any one ever had a small-pox pustule who had been thoroughly and *recently* vaccinated.

Dr. B. V. Willingham, of Lexington, Oglethorpe county, Ga., furnishes the following: During the prevalence of small-pox in January, February, and March, of the present year, he saw in Oglethorpe county, in the family of Mr. W., thirteen cases of variola and varioloid, (or small-pox modified by vaccination,) *four* of which died, neither one having been vaccinated; the remaining nine were vaccinated by himself, but having been previously exposed to the contagion of small-pox, developed light varioloid without any serious results. In another family, in the same neighborhood, a young man developed genuine small-pox, to which ten or twelve persons in the same house were exposed, but being promptly vaccinated no other case occurred. In another family of six they were all affected with the disease without previous vaccination, five of whom recovered; the vaccination made by himself upon one of the family, who had not developed the disease when he was called, manifested its influence about twenty-four hours previous to the appearance of the symptoms of small-pox—this case being manifestly modified, while the others were confluent. In another family he treated seven cases, four of whom died, (none being previously vaccinated,) and the remaining three, who recovered, were, in his judgment, unquestionably saved by the modifying influences of vaccination, which he instituted in time to ameliorate, though not to prevent, the disease. The three who lived, he remarks, were saved *solely* by vaccination. In two other families eleven cases were treated, with only two deaths—vaccination having modified a portion, the number not being stated. Among other facts of interest stated by him, was the occurrence of two cases among negroes, several miles apart, to which a large number of negroes were exposed, without any extension of the disease, doubtless due to the general vaccination he had performed in that vicinity.

The disease having also appeared in Clarke county, his services were required, and he informs us that, in one family, six cases occurred without a single recovery, none of whom were vaccinated; while three members of the family, who had been vaccinated and acted as nurses, entirely escaped. In another family, in the same county, he saw six cases without vaccination, four of whom died —the *nurses* having previously had small-pox, with one exception, where thorough vaccination had been performed, escaping alike.

All of the above, in Clarke county, with others referred to, but having no special interest, he states as having originated from exposure to the dead body of a lady who had died of small-pox, a large number of persons having attended the burial in ignorance of the disease with which she had died.

Dr. Willingham informs us that he has had several visitations of small-pox in his vicinity in the course of his professional life, during which he had attended a large number of cases, and could furnish a long list if it were necessary, adding to the evidence in favor of the protecting power of vaccination. He states that there had been an almost entire neglect of vaccination in some portions of the country to which he refers, and urges the importance of appealing to the legislature for the passage of a law compelling persons to have their children vaccinated at an early age.

We are under obligations to a distinguished gentleman, Dr. Jerome Cochran, of Mobile, Alabama, for a copy of a history of the small-pox epidemic in the city of Mobile, occurring during the latter part of the year 1874 and the earlier part of the year 1875, from which we obtain a number of interesting and valuable facts bearing upon the subject under consideration. He is unable to state with any accuracy the facts in regard to the date or the origin or the relations of the first cases to the subsequent epidemic, in consequence of the absence of competent sanitary administration, resulting from the want of appreciation, upon the part of the people and municipal authorities of Mobile of the importance of having the health of the city supervised by sanitary experts, the Mayor and Council having repealed their health ordinance, and dispensed, in the language of the writer, "almost contemptuously with the services of the board of health; and so it happened that no effort was made to extinguish the fires of infection until the pestilence had swelled into epidemic dimensions. At length, however, when the pestilence had already gained admission into every quarter of the city, and when the danger had assumed such portentous proportions as to make it impossible that it should be any longer overlooked, then, by common consent, the people appealed for help to the board of health, and the board of health, true to the unselfish spirit of the medical profession, placed themselves in the breach, and undertook to suppress the epidemic. The energetic action of the board of health met with appropriate reward. They undertook to relieve the city of small-pox, and did it. Let none think that it was an easy thing to do. It has been attempted often, but I know of no other instance, in which, under similar circumstances, anything like a similar success has been achieved. Often enough the first advances of a pestilence have been successfully resisted; but I know of no other instance, in which, after the epidemic was under full headway, and in the very season of the year most favorable for its rapid progress, it has been met and conquered." He states that small-pox has maintained its ground in New Orleans for five successive years, and during the present year has prevailed with as great a virulence as ever. The same general fact is said to exist also with regard to the city of New York, and he asserts with the most positive confidence that small-pox in Mobile did not die out *spontaneously*, but was overpowered and destroyed by agencies that were adopted and so energetically and perseveringly pursued by the board of health. These means in general terms were the vaccination of the entire population, (a large number never having been vaccinated), and as the effect of vaccination is not regarded as always permanent, the board advised all those who had been vaccinated before, to be revaccinated. They adopted such modes of disinfection as were known or regarded as destructive to the poison of small-pox, and by removing all sufferers, who could not be properly cared for, to the pest-house, and isolating others, they were enabled to accomplish the brilliant results so triumphantly detailed by the author of this history, who was also the executive officer of the board, and a conspicuous actor in this most creditable achievement. But such opportunities for the exhibition of professional and moral heroism are obtained at too great expense of human life. It were far better to have a record similar to the following, found in the Massachusetts Registration Report for 1868: "In Ireland vaccination was made compulsory in 1863. Since that period the Irish Poor Law Commissioners have carried out the provisions of the law, and the whole population has been vaccinated. The results are seen in the following figures, from which it appears that the Irish physicians have banished the small-pox from Ireland as St. Patrick is said to have banished the snakes: Whereas, in the periods 1830-40, 1840-50 and 1850-60, the respective annual average mortalities had been 5,800, 3,827 and 1,272, in the years 1864, 1865, 1867, 1868, they were 854, 347, 197, 20 and 19 respectively. In the first half of 1869 the whole number was three. The deaths from small-pox in Ireland since 1866 have been so few that it is fair to suppose the cases have been generally imported from abroad." Notwithstanding the wonderful protective power of vaccination in preventing the occurrence of small-pox, to provide against the ignorance, negligence and prejudices of certain portions of our people, we should not fail to guard every avenue, and to provide against all possible contingencies bearing upon the means of *prevention* by vaccination, but also those connected with its *extension*, when once introduced, as with all the possible care of legislators and medical men, there will probably be occasions calling for the use of means directed to the latter.

Sir James Simpson, of Edinburgh, Scotland, in his article entitled a "Proposal to Stamp out Small-pox and other Contagious Diseases," remarks: "In spite of the marvelous protective influences of vaccination, the mortality produced by small-pox in Great Britain is still very great and startling. If in any one year some overwhelming catastrophe destroyed all the living population of the counties of Nairn or Kinross, or swept away every living inhabitant of the cathedral cities of Lichfield, Ripon, or Wells, or slaughtered four or five regiments of soldiers, or smothered as many as five or six times the number of members of the House of Commons, such an event would assuredly appal and terrify the public and its guardians, and the strongest measures would no doubt be called for with the view of preventing the recurrence of the catastrophe, provided its prevention were at all possible. Is the similar amount of human slaughter to which our population is constantly subject by small-pox—not once, but continuously; not one year, but each year—preventible? I believe that it is so; and I believe further, that the hygienic measures required for effecting this prevention would be found neither specially difficult nor expensive to the country, while they would save annually hundreds, if not thousands, of our population from death by a disease which, even when it spares life, too often leaves permanent lesions and a broken and damaged constitution. To understand the means to which I point, let it be premised that small-pox is, like scarlet fever, measles, and hooping-cough, only a species of disease which, as a general law, attacks once in a lifetime, and is only propagated from an infected individual to a susceptible individual by a specific poison generated in the course of the malady, and transmitted from the affected to the healthy: first, by the near approach of the one to the other; secondly, by their contact; thirdly, by direct inoculation; or, fourthly, by families, or susceptible individuals, being exposed to the virus when it has been imbibed into clothes, etc., with which the sick have been in contact. We would no more expect this known species of disease, or poison, to originate *de novo* at the present day, under any combination of circumstances, than we would expect a known species of animal or plant—as a dog or a hawthorn—to spring up *de novo*, and without antecedent parentage." He states that the beneficial influence of Dr. Jenner's immortal discovery saved from death from small-pox in the then population in Great Britain (I believe about the year 1868) probably about eighty thousand lives yearly, and yet there were still about five thousand who died annually from this disease in the United Kingdom. Some among these five thousand had been duly vaccinated, and yet were susceptible of small-pox, after cow-pox, or vaccinia, just as persons are occasionally found susceptible of a second attack of small-pox after they have passed through a previous attack. Others were found susceptible in consequence of the vaccination having been imperfectly or inadequately performed, from various causes which are known to professional men who have investigated the subject, besides a number who had escaped any attempt at vaccination either from prejudice or neglect, or who happened to be exposed to the variolous poison antecedent to the age at which vaccination is usually performed. He admits that, by a more strict enforcement of the new compulsory laws of vaccination, and a greater amount of attention to its proper performance with reliable virus, the number of the susceptible class would be continually diminished. But believing in the possibility of completely "stamping out" the disease, as he terms it, he urges upon the authorities the importance of *isolation*, the methodic temporary seclusion, segregation, or quarantine, of those affected with small-pox until they have completely passed through the course of the disease and lost the power of infecting and injuring others. That some individual privation, inconvenience, and annoyance, would result from such a forcible measure as is here suggested is doubtless true, but that the State has not only the right, for the public good, thus to interfere with the liberty of the citizen will, we presume, be clearly established by a reference to the simple and striking illustration furnished by the law, in all civilized countries, to which allusion is made in this paper, which enforces the isolation of any individual affected with insanity,—be he rich or poor,—who is a homicidal lunatic, endangering the lives of others.

By a reference to the statutes of the State of Georgia in regard to the means for suppressing small-pox, it is ascertained that the legislation is of a wholly inadequate and indefinite character, and any action whatever upon the part of the authorities is, to a great extent, discretionary, and entrusted to those who, from various obvious causes, are not likely to be efficient in the administration of sanitary laws. For the accomplishment of the objects indicated in this paper, as well as for other sanitary interests of a vital character, it would seem to us important to complete the legislation of the last session of the General Assembly of the State, by which the State Board of Health was established, by a provision under the law, either directly or through the present board, for the organization of county Boards of Health throughout the State, who shall be auxiliary and subject to the present health authorities, and be fully empowered to enforce by all legitimate means such laws as sanitary science has determined as necessary to prevent the desolations of small-pox, as well as to have charge of all other matters pertaining to the public health within their respective counties. Whether this or some other mode of securing the object sought is adopted, it would seem to be clear that the State Board of Health ought to be empowered to enforce in some mode (not difficult to suggest) a thorough system of vaccination, by experts, throughout the State, and be permitted to protect communities, without regard to ordinary private rights, in times of public peril, arising from the appearance of the desolating scourge under consideration.

That there are imperfections, difficulties, and even dangers, connected with vaccination, will be readily admitted as not only necessary to a candid and truthful consideration of this subject, but for the purpose of defending it against the ignorance and irregularities to which its practice has been subjected, thus depriving it of its legitimate position as a perfectly safe and reliable means of preventing small-pox, in the hands of professional men, who should alone be allowed to use it. It is admitted as the result of long and careful observation that even when practiced with greatest care, the protection which is afforded by vaccination against small-pox is, in many instances, of a limited duration, the precise period of which has not been definitely determined, but is estimated by one of the most recent and distinguished writers upon the subject, (Heinrich Curschmann, Zeimssen's Encyclopedia,) varying from eight to twelve years, and it is recommended as necessary, or at least advisable, to secure all the immunity which vaccination is capable of yielding, that revaccination should be performed at something like this period throughout life. It is also doubtless true that a large portion of the uncertainty connected with the action of vaccine virus, in furnishing in all cases a permanent protection against small-pox, has been due to the unreliable character of the material used, as the result of the process having fallen into the hands of non-professional persons, who were incompetent to judge either of its character, effects, or the proper processes by which its action was to be obtained, and who had no stimulus of responsibility to secure diligent and faithful effort for obtaining proper results. Many persons suppose themselves to be protected by vaccination who have never been under its influence at all, and many others having been only partially affected by an imperfect vaccination with even good virus, have had their susceptibility to small-pox only partially removed, and are liable on exposure to contract varioloid, and, though escaping with their own lives, become the source of the propagation and perpetuation of the disease. The author just referred to, remarks that he ascertained to his entire satisfaction, with regard to one thousand small-pox patients whom he examined with reference to this point, that although they had been vaccinated, it had been done either improperly or too long before the date of the attack; not a single one coming up to the standard of a thorough and effective vaccination.

Besides the admitted uncertainties connected with vaccination as practiced by the people themselves, arising from perfectly obvious causes, and the want of absolutely permanent results in every case, even where the vaccine virus has been pure and the process practiced by an expert, we will acknowledge that many persons have been poisoned by the use, in ignorant or careless hands, of foul and decomposing animal matter, under the name of vaccine virus; and that after a long discussion the weight of evidence seems of late to be in favor of the possible transmission in rare cases of some other disease by the use of virus obtained from unhealthy persons.

When we compare the myriads of persons who have been vaccinated without any injurious results whatever, and who have thus been furnished with a perfect immunity against small-pox, with the small number in whom there is even a suspicion of having been injured thereby, we should only be stimulated to a more thorough investigation of the individuals and the causes of such exceptional manifestions, rather than to allow this infinite boon to humanity to be in any degree invalidated as regards not only its inestimable value, but its essential safety. Indeed, the possible transmission of this disease under such circumstances has only been established in regard to a single one, and with reference to this, it is well understood by medical men that such an accident would almost invariably result from ignorance or carelessness on the part of the operator. In connection with this subject we cannot too strongly suggest the necessity of urging upon our legislators the vital importance of establishing and maintaining some more efficient means than that now in operation for supplying the State with vaccine virus known to be reliable, and for the purity of which some one could be held responsible. Under the direction of intelligent County Boards of Health a large part of the work left undone by the carelessness or prejudices of the people in this particular could be accomplished in each locality coming under their supervision, provided they could be at all times supplied with pure virus, through a competent medical man at the seat of government; and in our judgment no more judicious or humane legislation could be adopted than a liberal appropriation of the people's money for an ample supply of this inestimable material, through such an agency as that suggested, to be distributed to County Boards gratuitously, and to which the medical profession in the State could look as a reliable source for a supply in any emergency. We have no means of forming even an approximate estimate, *from reliable data*, of the number of unvaccinated persons in the State of Georgia, but we think it can be no exaggeration to *affirm* that at least one-half of the entire population is thus unprotected; and if we were to draw our inferences from *facts* in certain localities where small-pox has prevailed, even this proportion of protected persons might, with good reason, be reduced one-half.

There are many interesting and important details involving vital questions, and most intimately connected with the practical execution of the plans suggested: such as the relations of small-pox to the season of the year; the comparative mortality of whites and blacks; the special means of disinfection; the vaccination of infants; the comparative value of humanized and bovine virus, and of the different modes of preserving it and inserting it; and many others, which would properly come under consideration, and could not be ignored in a strictly professional paper, but which would not be appropriate to the present occasion, or to the objects sought to be attained, and are therefore left untouched. There are other omissions, however, which we very much regret that we have not been able to supply, in the way of *statistics*, showing the absolute mortality from small-pox in the State, even in epidemics or outbreaks of the disease within the last year, and we specially regret the absence of statistics exhibiting the very large proportion of persons in Georgia who are believed to be entirely unprotected by vaccination. In either case, however, we have sufficient evidence to establish the imperative importance of early and decided legislation to secure large numbers of our people from this dreaded pestilence, and to make the accomplishment of this end one of the most important present objects of this board, securing which, and its beneficent results running down the years that are to come, it will erect for itself a monument more enduring than brass or stone.

J. P. Logan, M.D.,

H. H. Carlton, M.D.,

G. F. Cooper, M.D.,

*Committee.*

